# MgZnO High Voltage Thin Film Transistors on Glass for Inverters in Building Integrated Photovoltaics

**DOI:** 10.1038/srep34169

**Published:** 2016-10-10

**Authors:** Wen-Chiang Hong, Chieh-Jen Ku, Rui Li, Siamak Abbaslou, Pavel Reyes, Szu-Ying Wang, Guangyuan Li, Ming Lu, Kuang Sheng, Yicheng Lu

**Affiliations:** 1 Department of Electrical and Computer Engineering, Rutgers University, Piscataway, NJ 08854, USA; 2 Center for Functional Nanomaterials, Brookhaven National Laboratory, Upton, New York 11973, USA

## Abstract

Building integrated photovoltaics (BIPV) have attracted considerable interests because of its aesthetically attractive appearance and overall low cost. In BIPV, system integration on a glass substrate like windows is essential to cover a large area of a building with low cost. However, the conventional high voltage devices in inverters have to be built on the specially selected single crystal substrates, limiting its application for large area electronic systems, such as the BIPV. We demonstrate a Magnesium Zinc Oxide (MZO) based high voltage thin film transistor (HVTFT) built on a transparent glass substrate. The devices are designed with unique ring-type structures and use modulated Mg doping in the channel - gate dielectric interface, resulting in a blocking voltage of over 600 V. In addition to BIPV, the MZO HVTFT based inverter technology also creates new opportunities for emerging self-powered smart glass.

For decades, solar energy has been a leading technology in the search to replace fossil-fuel energy as a sustainable and clean energy source. However, cost and efficiency remain as major concerns^1^. As the price of photovoltaics (PV) modules has dropped, the inverters for energy conversion now count for more than 10% of the total cost of an entire PV system^2^. Additionally, in a conventional PV system all solar modules link to a central inverter; therefore, the overall system performance can be brought down by an underperforming module in the array, or by individual solar cells blocked from sunlight.

To solve this problem, and to optimize each individual solar panel, the micro-inverter technology^3^ has been proposed to embed inverters into each photovoltaic module. However, at the present time, the cost of micro-inverters is still higher than the cost of centralized inverters in small systems (≤10 kW)^4^. Another major factor hindering the wide adoption of solar energy is a conflict between aesthetics and energy saving. Many consumers find the placement of arrays of solar panels on buildings to be unsightly. Consequently, ways of integrating photovoltaics into buildings, i.e. Building Integrated Photovoltaics (BIPV) have been proposed^5^,^6^. BIPVs serve as building elements so that the appearance of houses won’t be compromised due to the post-installation of solar panels.

To address these two major issues, we have explored the possibility of creating new inverters which can be integrated with solar modules to form a photovoltaic system on glass (PV-SOG). In a PV-SOG, in addition to inverters, all other components including solar cells and controller circuits can be designed and fabricated using the same process. Therefore, not only the cost but also the unit size of a PV system is reduced^7^. The transparency and scalability of the PV-SOG’s exterior appearance make them attractive for application to BIPVs. Moreover, they possess better system reliability since every solar module is integrated with an individual inverter or inverter array. Our HVTFT inverter technology uses Magnesium Zinc Oxide (MZO) based wide band gap oxide semiconductor materials. Currently, the state-of-the-art high voltage and high power devices use the popular SiC and GaN transistors. However, both of these wide bandgap semiconductors require epitaxial growth at high temperature on strictly selected single-crystal substrates, which excludes their application in SOGs. In contrast, TFT technology made at low temperature has become a promising candidate for PV-SOGs. Several semiconductor materials have been tried out to make HVTFT devices. Amorphous Si and poly-Si HVTFTs have been studied since the 1980s^8^,^9^,^10^,^11^,^12^. Martin *et al*.^13^ utilized offset gate structure in amorphous Si TFT to operate devices under 400 V, and it provided on/off ratio ~10^6^. Chow *et al*. demonstrated amorphous Si TFTs, which can provide high voltage up to 800 V^14^; however, its poor performance (on/off ration ~10^4^) limits its application. In the case of poly-Si HVTFTs, Cheng *et al*.^15^ demonstrated poly-Si lateral double diffused metal oxide semi-conductor (LDMOS). The blocking voltage reached 240V, and on/off ratio was ~10^6^. Jamshidi-Roudbari^16^ *et al*. reported a multi-gate poly-Si HVTFTs which provided high current (~50mA) and high on/off ratio (~10^8^) for actuator application. However, the bias condition (V_GS_~30V, V_DS_~35V) is not high enough for inverter application. Overall, poly-Si HVTFTs show better driving capability; however their low blocking voltage and non-uniformity from grain boundaries make them inadequate to meet the requirements of PV-SOG. In addition, Si-based TFT technology suffers from the absorption of visible light, restricting its application for transparent electronics. Organic TFTs which offer low cost and low process temperature have been used in display and RFID technologies. Recently, Smith *et al*. reported^17^ a high-voltage organic thin-film transistor (HVOTFT), which shows switch drain-to-source voltages higher than 300 V with a controlling voltage range from 0 to 20 V. However, its low mobility, poor long-term stability, and the degradation at higher temperatures basically exclude its application in PV-SOGs, which operate under sunlight radiation, and its lifetime, like the regular residential solar cells, is expected to be more than 25 years.

Since Nomura *et al*.^18^ demonstrated Indium Gallium Zinc Oxide (IGZO) TFTs with high electrical performance at low process temperature, oxide semiconductor TFTs have emerged in many applications, especially in displays and transparent electronics. In the area of HVTFT, Jeong *et al*.^19^ reported an IGZO HVTFT, which operated at above 100 V with an on/off current ratio of 10^7^. Although this is beyond the regular operating voltage in regular TFTs, it is still not sufficient to be used in inverters for a solar PV system. Furthermore, it is desired to use indium-free materials due to the high cost of indium, especially in the case of large-area electronic systems such as solar cells. In addition, the toxicity of IGZO due to its high indium concentration is of considerable environmental concern.

In this work, we report a Mg_0.03_Zn_0.97_O (MZO) based HVTFT built on a transparent glass substrate. ZnO possesses a wide energy bandgap (~3.34 eV at room temperature), similar to SiC and GaN. As a TFT channel material, ZnO can sustain a high electrical field^20^,^21^, which is particularly important for high voltage devices, such as inverters. The thermal conductivity of ZnO is as good as Si^22^. ZnO and MZO have been used for various solar cells^23^,^24^; thus, MZO inverters would benefit PV-SOG technology as various components including cells, control devices, and inverters can be integrated on the same glass substrate. However, TFTs using pure ZnO as a channel suffer from threshold voltage shift and thermal instability^25^,^26^, which would hinder its use as an inverter. To keep intrinsic advantages but resolve the instability accompanied with pure ZnO, we have introduced a small amount of Magnesium (Mg) into ZnO to form a ternary Mg_x_Zn_1−x_O (MZO) as the TFT channel material which suppresses oxygen vacancy in the channel layer. In addition, the interface between the gate dielectric (SiO_2_) and the MZO channel is specially designed by modulation-doping Mg into MZO to significantly increase the TFT blocking voltage. This MZO based HVTFT on glass technology naturally fits the PV-SOGs, which are not only suitable for BIPV but also promising for the self-powered smart glasses^27^. It may also create opportunities for other high voltage applications, such as high dc voltage sensing^28^, high-speed printers^29^, flat-panel x-ray imaging systems for medical radiology^30^, and space engineering^31^.

## Results

### Device structure

**Figure 1:**
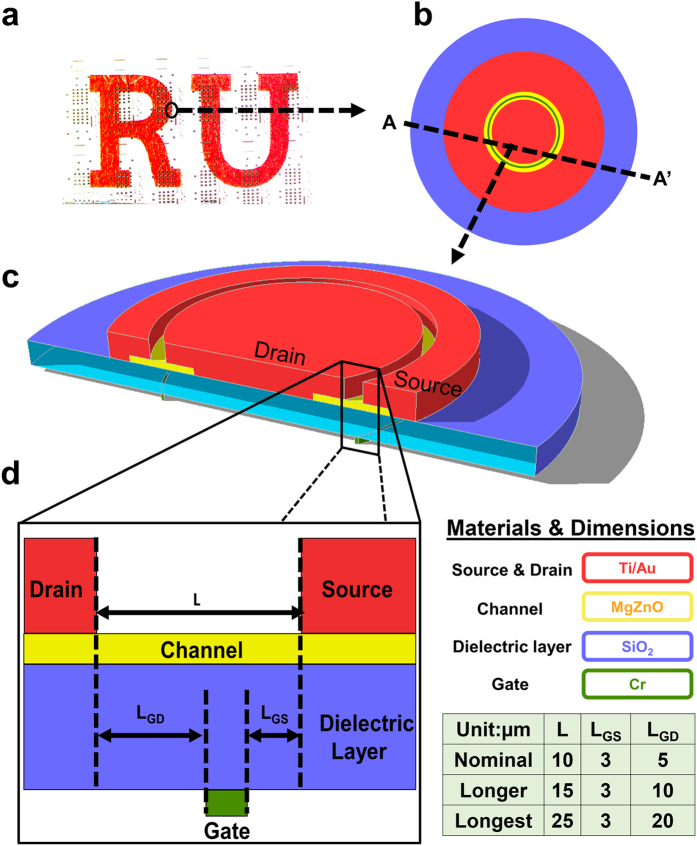
The overview of the structures of HVTFTs. (**a**) A photograph displays the HVTFTs on a transparent glass substrate. Schematic diagrams of an MZO HVTFT with a ring structure are shown: (**b**) the top view and (**c**) the three-dimensional cross-sectional structure along A-A’ in (**b**,**d**) the dimensions and structure of a single HVTFT device.

The regular TFT has a rectangular channel. For an HVTFT, such a design would introduce non-uniform electrical field distribution with the highest field located at the corners of the channel, which limits the blocking voltage of the devices. To solve this problem, a ring structure is designed as shown in Fig. 1. The electrical field distributions of the ring and the rectangular structures are included in the supporting information. From the comparison between these two configurations, it can be seen that the field distribution from drain to source in the ring structure is much more uniform and the highest field is ~50% less than in the rectangular counterpart. The HVTFT has a bottom gate inverted-staggered configuration, and it includes two offset regions: gate to drain and gate to source, respectively. For comparisons, three types of materials were deposited on a SiO_2_ dielectric layer, serving as the channel layer with a thickness of 50 nm: (i) pure ZnO, (ii) Mg_0.03_Zn_0.97_O (MZO), and (iii) Mg_0.03_Zn_0.97_O plus a modulation-doped thin layer (m-MZO). In the m-MZO HVTFT, a modulation-doped 10 nm Mg_y_Zn_1−y_O transition layer (MZO-TL) is inserted between the MZO channel layer and the SiO_2_ dielectric layer, and the Mg composition (y) in the Mg_y_Zn_1−y_O TL decreases from the side adjacent to SiO_2_ (y = 1) to the other side adjacent to the channel (y = 0.03).

### Transfer characteristics and thermal stability

**Figure 2:**
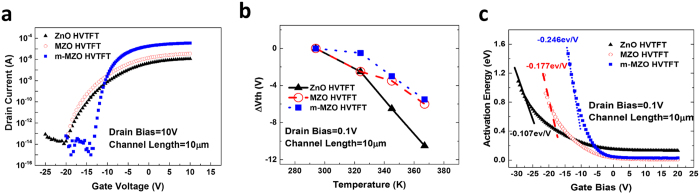
The electrical performances of HVTFTs. (**a**) Transfer characteristics of ZnO, MZO and m-MZO HVTFTs with V_DS_ = 10V. (**b**) The shift of threshold voltage of HVTFTs at different temperatures. (detail definition of the shifts of threshold voltage can be found in the Supplementary Information.) (**c**) The extracted activation energy of drain current as a function of gate bias in HVTFTs.

The transfer characteristics of HVTFTs with three different channel materials and structures are shown in Fig. 2a. Compared with ZnO HVTFT, MZO HVTFT shows a better subthreshold slope (S.S.) and on-current. The most significant improvement of the MZO over the pure ZnO channel layer is in its thermal stability, which is one of the critical requirements for high voltage devices being integrated into PV-SOGs. By measuring the transfer characteristics of HVTFTs at different temperatures, the shifts of threshold voltage (ΔV_th_) are compared in Fig. 2b, and the detail definition of the shifts of threshold voltage can be found in the Supplementary Information. As temperature increases from 294 K (room temperature) to 367 K, MZO HVTFT only shows a threshold voltage shift ∆V_th_ of −6 V in comparison to ∆V_th_ of −10.5 V in the pure ZnO counterpart. This negative shift of threshold voltage at higher temperatures results from the thermally activated electrons from the trap states located in the channel and in the interface between the channel and gate dielectric SiO_2_.

In Fig. 2c, the activation energy of drain currents at different gate biases is extracted from the Arrhenius plot (*ln I*_*D*_ *vs. T*^−*1*^) in equation (1):


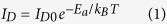


where *I*_*D0*_ is the drain current constant, *k*_*B*_ the Boltzmann constant, *T* the temperature in Kelvin, and *E*_*a*_ the activation energy of drain current.

If the trap density in TFT is high, the moving rate of the Fermi level with respect to the gate bias from the deep level to the conduction band is roughly inversely proportional to the total trap density. The steeper falling rate (0.177 eV/V) of the MZO HVTFT, as opposed to that of the ZnO HVTFT (0.107 eV/V), indicates that the MZO TFT has a lower trap density. Because both MZO and ZnO HVTFTs are fabricated on the same SiO_2_ gate dielectric layer, the interface trap densities of the two devices are similar. Therefore, the improvement of thermal stability in MZO HVTFT over ZnO HVTFT is mainly attributed to the reduction of traps in the bulk channel. In fact, the Mg-O has a stronger bonding than Zn-O^25^, resulting in the lower density of oxygen vacancy in the MZO channel than that of the pure ZnO channel.

In the comparison between MZO HVTFT and m-MZO HVTFT, it is found that the later shows an order higher on-current and a steeper S.S. than the MZO counterpart. The steepest falling rate (0.246 eV/V) of the activation energy of the drain current suggests a nearly 40% lower total trap density in m-MZO HVTFT than in MZO HVTFT. Since both of MZO and m-MZO HVTFTs are made up of the same MZO channel and SiO_2_ gate dielectric layer, these differences in characteristics between the two HVTFTs are mainly caused by the different interface properties between the channel and gate dielectric layer. Specifically, the unique interface design and engineering using modulation doping of Mg in m-MZO HVTFT reduces the interface trap density. Therefore, the total trap density in m-MZO HVTFT is lower than in the MZO HVTFT.

### Mg_y_Zn_1−y_O transition layer (MZO-TL) as a diffusion barrier

**Figure 3:**
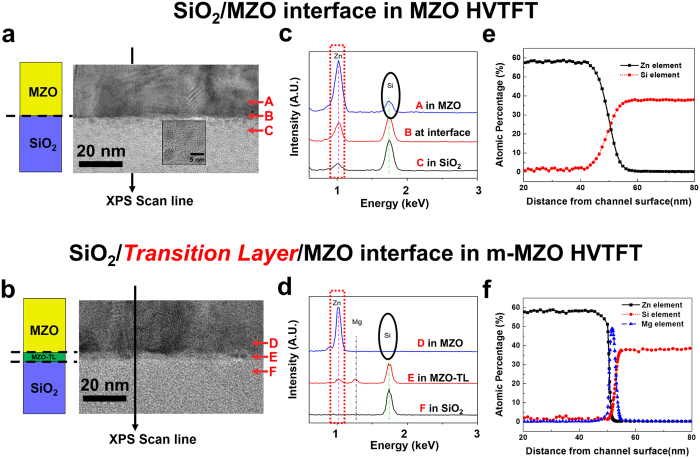
Material characterization near the interfaces. TEM images of the interfaces between the channel layer and the SiO_2_ dielectric layer (**a**) without a transition layer (for MZO HVTFT) and (**b**) with a modulation doped transition layer (for m-MZO HVTFT). The inset of Fig. 3(a) features the gray dots found in the SiO_2_ layer in the MZO sample. EDS spectra of Zn, Mg and Si elements at the different locations (marked in the TEM images) across the channel – gate dielectric interface for (**c**) MZO and (**d**) m-MZO samples. Depth profiles of atomic percentage of Si, Zn, and Mg from XPS measurements of (**e**) MZO and (**f**) m-MZO samples along the scan lines shown in the TEM pictures. The oxygen profile is not included.

To understand the effect of the MZO-TL on improving the properties of the interface between the channel and gate dielectric layer, the cross sections of HVTFTs prepared by a Focus Ion Beam (FIB) were studied using a transmission electron microscopy (TEM). Figure 3a and bshow the images of the interface regions in MZO and m-MZO HVTFT samples, respectively. As shown in the inset of Fig. 3a, the gray dots are observed only in the SiO_2_ dielectric layer of the MZO HVTFT sample. To identify these dots, energy-dispersive X-ray spectroscopy (EDS) was used to analyze the elemental composition of the films at different positions (point “A” to “F”) across the interface. As shown in the dashed box of Fig. 3c, the Zn peak in MZO HVTFT appears not only in the MZO channel area (point A) but also near the interface (point B) and even inside the gate dielectric SiO_2_ layer (point C). The EDS results further confirm that the observed gray dots are related to the Zn element. Moreover, in the EDS spectrum, there is a Si peak marked by the “circle” in the MZO area (point A), The results indicate that the interdiffusions occur across the interface (point B): Zn diffuses from the MZO layer into SiO_2_ while Si diffuses from the SiO_2_ layer into the MZO layer. In contrast, for m-MZO HVTFT, as shown in the dashed box of Fig. 3d, the Zn peak appears in the MZO area (point D), only a tiny peak appears near the interface (point E), but no Zn peak is observed inside the SiO_2_ layer (point F). On the other hand, as shown in the circle, there is no Si peak detected in the MZO area (point D). Therefore, the modulation-doped thin transition layer inserted between the MZO and SiO_2_ acts as a diffusion barrier, which hinders Zn and Si diffusion across the interface between the channel and gate dielectric layer.

X-ray photoelectron spectroscopy (XPS) was used to estimate the atomic percentages of different elements in the interface regions. The depth profiles were obtained by using the *in-situ* sputtering process. Figure 3e,f show depth profiles of atomic percentages of Si, Zn, and Mg in the MZO and the m-MZO HVTFT, respectively. A small amount (3%) of Mg doping is barely shown inside the MZO channels in both of MZO and m-MZO HVTFT samples due to the detection limit of XPS. However, a narrow peak of Mg does appear in the m-MZO HVTFT, produced from the Mg_y_Zn_1−y_O transition layer (MZO-TL). In the MZO sample, Zn diffuses extensively into the SiO_2_ layer as indicated by the length of overlapped XPS profiles of SiO_2_ and MZO. In contrast, there is an abrupt interface with negligible overlapped profiles of Si and Zn in the m-MZO sample. TEM/EDS and XPS characterizations provide consistent results that the phenomenal interdiffusion between the SiO_2_ gate dielectric layer and the MZO channel layer is only detected in the MZO HVTFT. The MZO-TL in m-MZO HVTFT acts as a diffusion barrier, which effectively blocks the interdiffusion of Zn from the MZO to SiO_2_, as well as Si from SiO_2_ to the MZO channel.

### High voltage blocking capability

**Figure 4:**
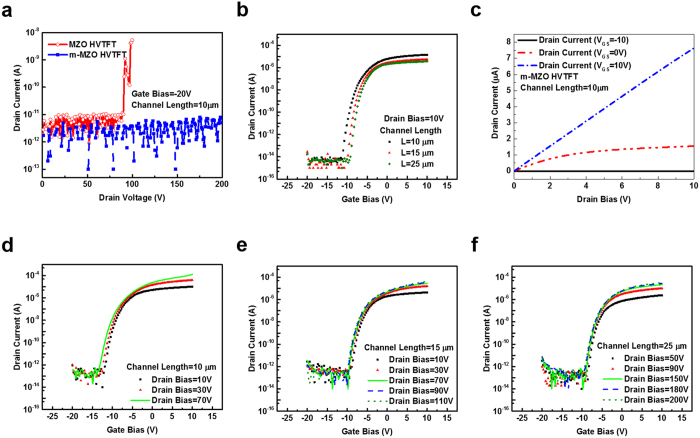
The electrical performances at high bias conditions and the blocking capabilities of HVTFTs: (**a**) drain leakage current of off state in MZO and m-MZO HVTFTs; (**b**) transfer characteristics of m-MZO HVTFTs with different channel lengths (the detailed dimensions of m-MZO HVTFTs are listed in the Table of Fig. 1d.); (**c**) the output characteristics of m-MZO HVTFT with a channel length L = 10 μm; (**d–f)** the transfer characteristics at high bias conditions of m-MZO HVTFTs with a channel length L = 10, 15, and 25 μm, respectively.

Having the capability of blocking high voltages and operating at high bias conditions reliably are the essential characteristics for the application of high voltage transistors in PV inverters. Since ZnO HVTFT fails to show thermal stability, we here only compare MZO and m-MZO HVTFTs. The results of blocking voltages of MZO and m-MZO HVTFTs with the same channel length (L = 10 μm) are shown in Fig. 4a. The drain leakage current of MZO HVTFT increases abruptly, and the device burns down at V_DS_ = 90 V. In contrast, the drain leakage current of m-MZO HVTFT keeps as low as 10^−12^ A even at much higher V_DS_ = 200 V (here 200 V is the limitation of HP-4156C used for testing).

A comparison of the transfer characteristics at normal bias (drain bias = 10 V) among three m-MZO HVTFTs with different channel lengths is presented in Fig. 4b. There is a trade-off between blocking capability and driving capability in m-MZO HVTFT. As the channel length increases, the blocking voltage increases; however, the on-current drops. The values of the blocking voltage/on-current for the nominal (L = 10 μm), longer (L = 15 μm), and longest (L = 25 μm) m-MZO HVTFT are 300 V/3.5 × 10^−5^ A, 427 V/6.61 × 10^−6^ A, and 609 V/4.57 × 10^−6^ A, respectively. The output characteristics of m-MZO HVTFT of 10 μm are presented in Fig. 4c. It shows better saturation behavior at low gate bias. At high gate bias, the drain current increases as the drain bias increases. This kink effect was also observed in the IGZO HVTFT^19^. It might be related to the channel length modulation induced self-heating effect^19^,^32^.

The m-MZO HVTFTs with channel length of 10, 15, and 25 μm have the highest operating drain bias of 70, 110, and 200 V as shown in Fig. 4d–f, respectively. The maximum drain voltage without degrading the on-current defines the highest operating drain bias of each HVTFT, except for the case of 25 μm which only shows 200 V due to the limitation of the testing equipment. Under these high drain bias conditions, all m-MZO HVTFTs show a high on/off ratio of more than 10^7^, and the V_OFF_ and off-current almost constant at any drain bias condition. Theses features indicate that m-MZO HVTFTs are stable even under high bias conditions. The m-MZO HVTFT of 25 μm can operate at drain bias of 200V with a blocking capability over 600 V, suitable to be used as an inverter of PV-SOG.

## Discussion

The interface engineering using a modulation-doped thin MZO transition layer (MZO-TL) in the m-MZO HVTFT improves transfer characteristics, thus enables high voltage blocking capability. Such improvements are mainly attributed to the prevention of Zn diffusion into the SiO_2_ dielectric layer. Zn could diffuse as ions, such as Zn^2 + ^into the dielectric layer and then become the fixed charges in SiO_2_. Because the positive Zn^2 + ^trapped inside SiO_2_ would attract electrons, it requires an extra negative gate bias voltage to deplete the channel. As a result, MZO HVTFT, which has extra diffusion of Zn^2 + ^into SiO_2_, has more negative V_OFF_ than m-MZO HVTFT does. Moreover, the out-diffusion of Zn from the MZO channel layer would generate Zn-related defects, such as Zn vacancies and Zn interstitials in the MZO channel layer, especially near the MZO/SiO_2_ interface, thus degrading the electrical performance of transistors. The total trap density from the subthreshold slope (S.S.) can be estimated in the equation (2)^33^:


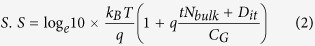


where *q* is the elementary electric charge, *k*_*B*_ the Boltzmann constant, *T* the temperature in Kelvin, *t* the channel thickness, *N*_*bulk*_ the bulk trap density, *D*_*it*_ the interface trap density, and *C*_*G*_ the capacitance per area of the gate dielectric layer (*C*_*G*_ is 1.73 × 10^−4^ F/m^2^ and 1.69 × 10^−4^ F/m^2^ for MZO and m-MZO HVTFT, respectively. The theoretical values of SiO_2_ = 3.9 and MgO = 9.90^34^ are used in estimation). The total trap density includes the bulk trap (*tN*_*bulk*_) and the interface trap (*D*_*it*_), which are calculated to be 2.14 × 10^12^ cm^−2^ and 8.36 × 10^11^ cm^−2^ for MZO and m-MZO HVTFT, respectively. Since the channel material is the same, the difference in the total charge density between HVTFT and m-HVTFT is approximately equal to the reduction of the interface trap density, which is 1.3 × 10^12^ cm^−2^. By adding a modulation doped transition layer into the m-MZO HVTFT, Zn diffusion into the SiO_2_ dielectric layer is significantly suppressed. The interface engineering successfully adjusts the threshold voltage V_th_ close to 0 V and makes the S.S. steeper than that of MZO HVTFT.

**Figure 5:**
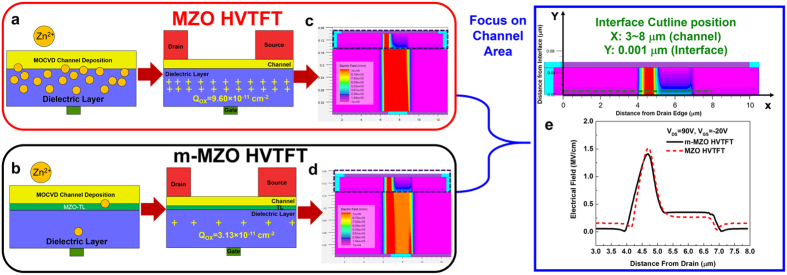
The simulation results of electrical field distribution in HVTFTs. Schematic diagrams of different interface designs and layer structures of (**a**) MZO and (**b**) m-MZO HVTFT, respectively. The SILVACO simulation results on two-dimensional distributions of the critical electrical field in the nominal (L = 10μm) channels of (**c**) MZO HVTFT and (**d**) m-MZO HVTFT, respectively. (**e**) A comparison of the electrical field of MZO HVTFT and m-MZO HVTFT. The cutline of the electrical field is near the interface of channel/gate dielectric and above the gate edge where the maximum electrical field locates.

The addition of the MZO-TL also enables the higher blocking voltage. The ideal breakdown field of a pure MgO is 12 MV/cm^35^. Although the effective thickness of the dielectric layer increases by adding the MZO-TL, a 10 nm MZO transition layer can not provide more than 12 V of the blocking voltage. Furthermore, the gate leakage current keeps at a similar level in both HVTFTs after the breakdown at high drain bias. Therefore, the significant enhancement of the blocking voltage in m-MZO HVTFT cannot be attributed to the extra voltage drop on the MZO-TL. In order to understand the fundamental cause of the improvement in blocking voltage, SILVACO software was used to simulate the electric field distributions in the two different devices. As drawn in the Fig. 5a, MZO HVTFT possesses extra positive oxide charges in comparison to m-MZO HVTFT. The amount of the extra equivalent oxide charges per unit area (Q_OX_) is estimated based on the equation (3):


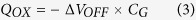


where C_G_ is the capacitance per area of the gate dielectric layer. The difference in V_OFF_ between MZO and m-MZO HVTFT is around −6V, so the extra 6.47 × 10^11^ cm^−2^ of positive charges are placed in the MZO HVTFT. The impact of oxide charges near the interface on electrical field distribution in the device is shown in Fig. 5. The more detailed simulation, including the consideration of the gate connection between the gate ring and the probing pad can be found in Supplementary Information. The maximum values of the electrical field near the interface are 1,520 kV/cm and 1,410 kV/cm for MZO and m-MZO HVTFT, respectively. The reduction of the maximum electrical field allows m-MZO HVTFT to operate at higher drain bias, enabling higher blocking voltage. Overall, the MZO-TL in m-MZO HVTFT acts as a barrier against Zn diffusion so that the interface states and trapped charges are reduced. This improvement leads to the decrease of the maximum electrical field near the channel-gate dielectric interface, resulting in the increase of the blocking voltage.

In summary, we have demonstrated the MZO high voltage thin film transistors on a transparent glass substrate. The ring structure design reduces the electric field crowding effect in the devices. The thermal stability is enhanced by doping ZnO with a small amount of Mg to form the MZO channel layer. The interface design and engineering are conducted by inserting a modulation-doped ultra-thin MZO transition layer between the SiO_2_ gate dielectric layer and the MZO channel, and they significantly improve the subthreshold slope and on-current values. More importantly, it enables high blocking voltage of 609 V with an on/off ratio of 3.3 × 10^8^, and operating voltage over 200 V. The comprehensive characterizations confirm that the enhancement in the HVTFT performance is mainly attributed to the reduction in interface trap density and trapped charges, which leads to the reduction of the maximum electric field in the channel. This MZO-based HVTFT on glass technology is promising to serve as the solar inverter in PV-SOG technology to implement the emerging BIPV and self-powered smart glass

## Methods

### Material Preparation and Device Fabrication Process

The HVTFTs were fabricated on 0.4 mm thick commercial glass substrates. A 50 nm chromium (Cr) layer was deposited by sputtering, and patterned using a dry etching process to serve as the bottom gate electrode. Then, a 200 nm SiO_2_ layer was deposited by plasma enhanced chemical vapor deposition (PECVD) as the gate dielectric layer. Following the SiO_2_ deposition, the channel was deposited using metal organic chemical vapor deposition (MOCVD) at 400 °C. DeZn (diethyl zinc) and MCp2Mg (bis (methylcyclopentadienyl) magnesium) were used as the precursors for Zn and Mg, respectively. Three types of channel layers (thickness of 50 nm) were deposited on SiO_2_: (i) pure ZnO, (ii) Mg_0.03_Zn_0.97_O (MZO), and (iii) modulation-doped Mg_0.03_Zn_0.97_O (m-MZO). In the m-MZO HVTFT, a 10 nm modulation-doped Mg_y_Zn_1−y_O transition layer (MZO-TL) was inserted between the Mg_0.03_Zn_0.97_O (MZO) channel layer and the SiO_2_ dielectric layer, and the Mg composition (y) in the TL decreases from the side adjacent to SiO_2_ (y = 1) to the other side adjacent to the channel (y = 0.03). The source and drain metallization (100 nm titanium (Ti)/50 nm gold (Au)) was deposited using electron beam evaporation, followed by a normal lift-off process. A photoresist film was coated on top of the TFT channel, serving as a passivation layer to prevent ambient absorption/desorption during electrical testing. HVTFTs with three different channel lengths are listed in the table of Fig. 1d. The channel lengths/gate-to-drain offset lengths are 10/5 μm, 15/10 μm, and 25/20 μm for nominal, longer, and longest HVTFT, respectively. The gate-to-source offset is kept the same of 3 μm.

### Testing Conditions

The electrical measurements under the low bias were conducted using an HP-4156C with an HP-41501B Pulse Generator. With the boost from the connection of a pulse generator, the maximum voltage of the HP-4156C electrical testing system was limited to be 200 V. The system which had a current resolution of 1 × 10^−15^ A was used for all transfer characteristics. For electrical measurements under high bias, a high voltage testing system was built based on a Tektronix 370 with the probe station. As the current resolution of Tektronix 370 only reached 1 × 10^−6^ A, it was only used for the testing of blocking voltages. In order to avoid problems with arcing and tracking due to environmental conditions, the devices were immersed in Fluorinert FC-40 during the high voltage measurements. The electrical measurements at different temperatures were conducted using an Agilent 1500B. All measurements were conducted in a light-tight probe station.

The samples used in the material analysis were prepared under the same device process except that the materials samples did not go through patterning and metallization processes. The structural and interfacial properties were analyzed using JEOL 2100F Field Emission Lorentz Transmission Electron Microscopy (TEM), Energy-dispersive X-ray spectroscopy (EDS), and X-ray photoelectron spectroscopy (XPS). The TEM samples were prepared by using a FEI HELIOS 600 Dual Beam Focus Ion Beam (FIB).

## Additional Information

**How to cite this article**: Hong, W.-C. *et al*. MgZnO High Voltage Thin Film Transistors on Glass for Inverters in Building Integrated Photovoltaics. *Sci. Rep.* **6**, 34169; doi: 10.1038/srep34169 (2016).

## Supplementary Material

Supplementary Information
