# Supplementary material for: MgZnO High Voltage Thin Film Transistors on Glass for Inverters in Building Integrated Photovoltaics
Source: Sci Rep. 2016 Oct 10;6:34169. doi: 10.1038/srep34169 (PMC5056374; doi:10.1038/srep34169)
Supplement: Supplementary Information [file srep34169-s1.pdf]

Supplementary Information

MgZnO High Voltage Thin Film Transistors on Glass for Inverters in Building Integrated Photovoltaics

Wen-Chiang Hong, [1] Chieh-Jen Ku, [1] Rui Li, [1] Siamak Abbaslou, [1] Pavel Reyes[1], Szu-Ying Wang[1], Guangyuan Li[1], Ming Lu, [2] Kuang Sheng, [1,3] and Yicheng Lu [1]*

[1] Department of Electrical and Computer Engineering, Rutgers University, Piscataway, NJ 08854, USA

[2] Center for Functional Nanomaterials, Brookhaven National Laboratory, Upton, New York 11973, USA

[3]Currently with College of Electrical Engineering, Zhejiang University, Hangzhou 310027, China,

*Corresponding author: Phone: (848) 445-3466; E-mail: ylu@rci.rutgers.edu

Statistical Data of Electrical Performance

The statistics of electrical performances of m-MZO HVTFTs are presented in Figure S1, including (a) on-current, (b) off-current, and (c) blocking voltage. The data show the trade-off between blocking voltage and on-current; furthermore, such trade-off is directly affected by the offset length. The dominate factor of the variation in data is attributed to the device processing issues, especially the mask misalignment in the photolithorgraphy process of the ring-structures. Such variation could be suppressed by refining the photo-mask design and using a better alignment tool. It is noticed that the variation of the off-current (Figure S1b) is larger than that of on-current (Figure S1a). This is due to much smaller values of the off-current which are close to the measurment limit of the instrument system.

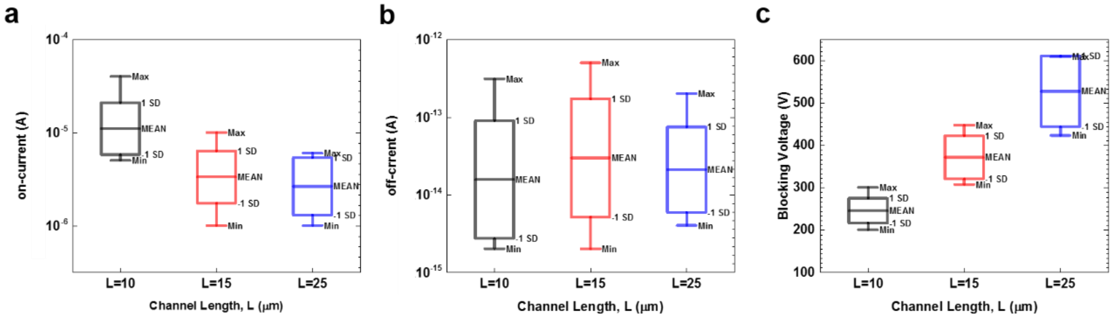

Figure S1. The statistic data of (a) on-current, (b) off-current , and (c) blocking voltage of the m-MZO HVTFTs with three different channel lengths (L = 10, 15, and 25 μm). The error bar provides mean , maximum (Max), minimum (Min), plus one standard deviation (1SD) and minus one standard deviation (-1SD) values. The numbers of data points are taken from 25 , 25, and 15 devices with the channel length of 10, 15, and 25 μm.

Simulation of Electrical Field Distribution

(i) Comparison between ring and rectangular configurations

SILVACO software was used to simulate the electrical field distribution in TFT devices with the ring (Figure S2a) and rectangular (Figure S2b) configurations, respectively. For comparison, the m-MZO HVTFTs with both configurations that have the equivalent channel length and offset region length are used in the simulation. As shown in Figure S2d, at ON state of high voltage operation, the electrical field crowding occurs on the drain side around the corner of the rectangle where the maximum field reaches over 2,800 kV/cm. This becomes the weak point where the breakdown of TFT happens. On the contrary, the field distribution in the ring structure (Figure S2c) is uniform from drain to source, and the highest field is 1,420 kV/cm, which is approximately 50% less than in the rectangular counterpart. It shows that the symmetric design of a ring structure removes the severe electrical field crowding around the corners of a rectangular channel. Therefore, the HVTFT with the ring structure is able to work at a higher bias voltage and offers a higher blocking voltage over the regular rectangular counterpart.

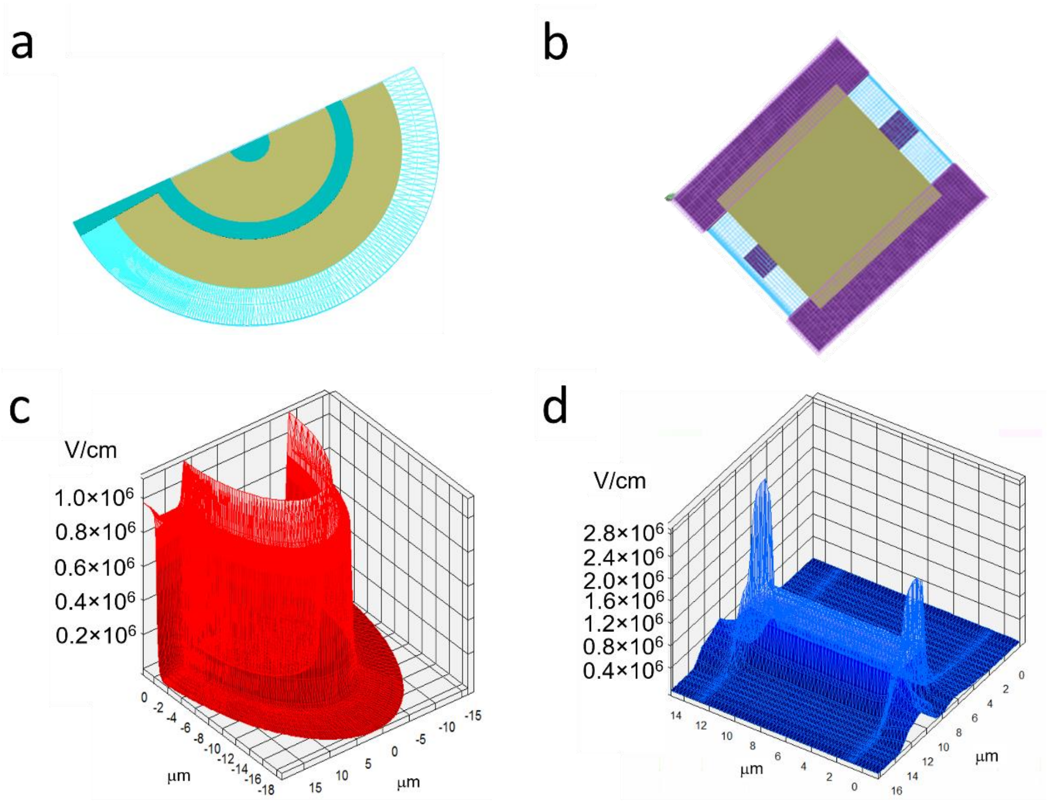

Figure S2. Top view of (a) a HVTFT with a ring structure and (b) a HVTFT with a rectangular structure. Simulation results of the electrical field distribution in the HVTFT channel (c) with a ring structure and (d) with a rectangular structure.

(ii) Comparison between MZO HVTFT and m-MZO HVTFT

In the ring configuration, a contact pad is needed to connect the gate as shown in Figure S3a. The electrical field in the gate connection area is different from the other area in the ring. In order to analyze the influence of the gate connection, the simulation was also conducted for the area with the gate connection. As shown in the Figure S3b, the “Path 1” is the majority of areas without the gate connection, and the “Path 2” is the area with the gate connection. The cross-section view of structures of the “Path 1” and “Path 2” are shown in Figure S3c. The electrical

field distributions in "Path 1" and "Path 2" in MZO HVTFT and m-MZO HVTFT are simulated and the results are presented in Figure S4. In both "Path 1" (Figure S4a) and "Path 2" (Figure S4b) regions, m-MZO HVTFTs show the lower maximum electric field than that of MZO HVTFTs. It indicates that the transition layer enables to reduce the peak electrical field in the devices with the gate connection.

The effects of the gate connection on the electrical field of HVTFTs along the cutlines are shown in the Figure 5. For both of m-MZO and MZO HVTFTs, the distribution and maximum value of electrical fields in the path 1 and 2 are almost the same. The major difference is the electrical field in the gate-source offset region. In the path 1, the electrical field drops quickly; on the contrary, in the path 2 the electrical field is constant in whole gate-source offset region.

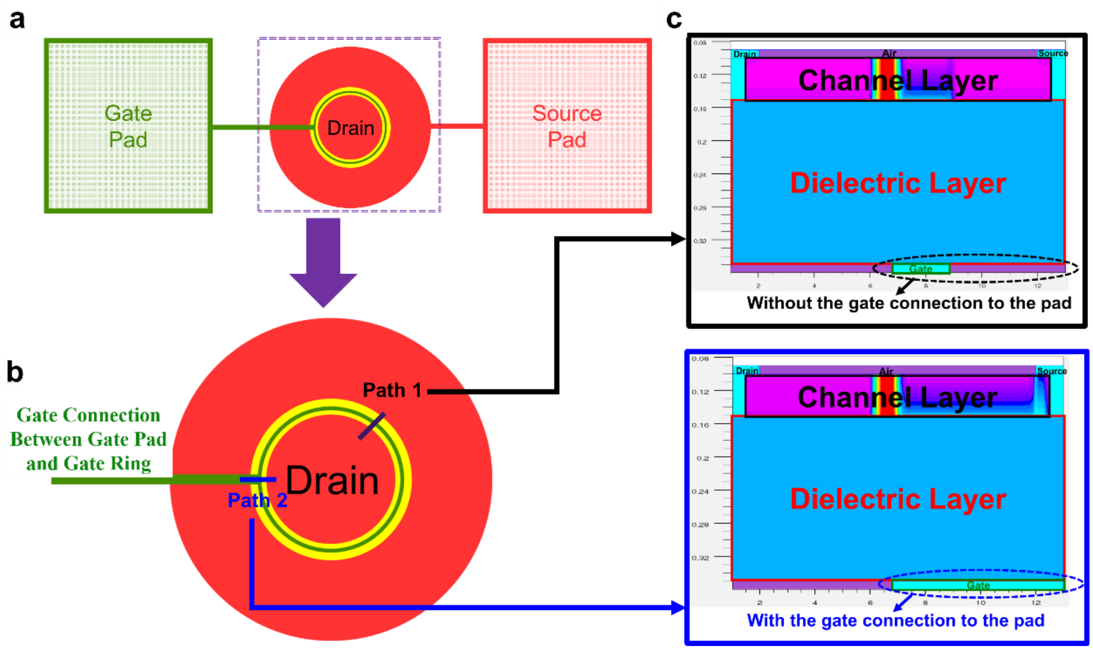

Figure S3. The schematics of 2D simulation areas of a HVTFT with and without the gate connection: (a) top-view of the whole device, (b) two simulation paths, and (c) cross-sectional view of structures along two paths

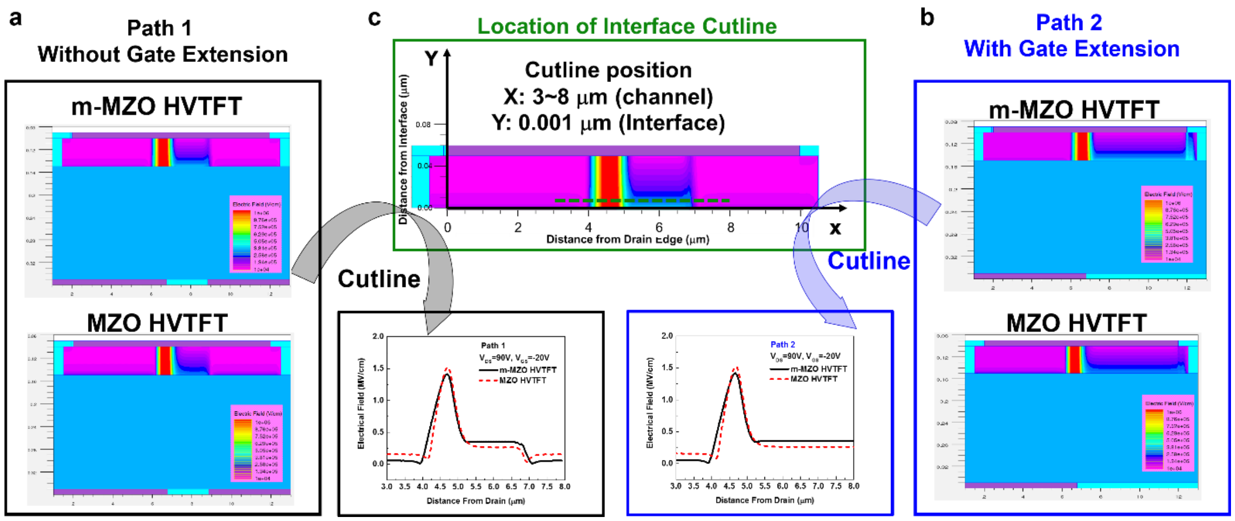

Figure S4. The simulation results of the electrical field distributions in the channel layers. The comparisons of electrical field distribution between MZO and m-MZO HVTFTs are presented for the configuration: (a) without the gate connection and (b) with the gate connection. (c) The location of 1D cutline. The comparison between m-MZO and MZO HVTFT along Path 1 and Path 2 are shown.

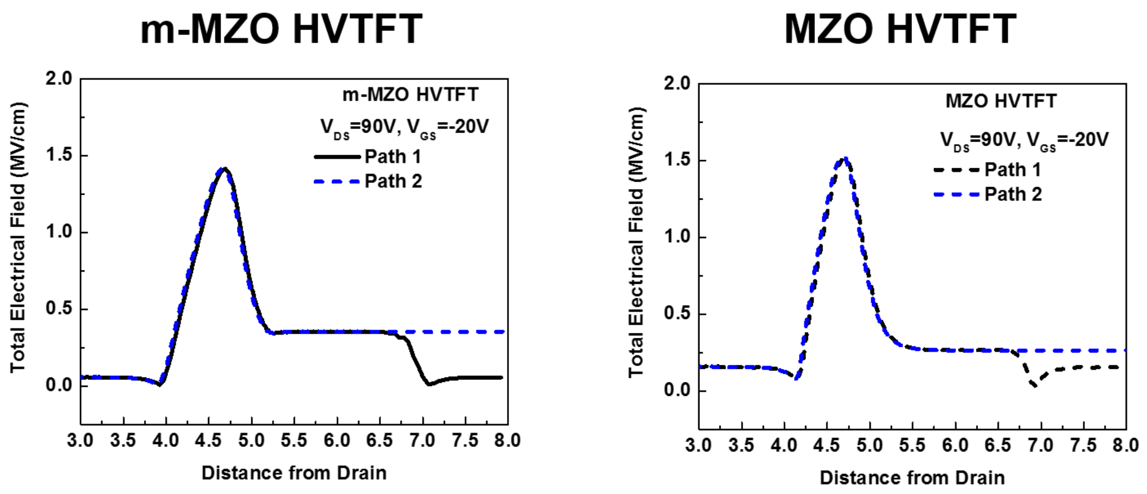

Figure S5. The influence of the gate connection (Path 1: without the gate connection; Path 2: with the gate connection) to the electrical field distribution at the interface cutlines of m-MZO and MZO HVTFTs.

Definition of Electrical Parameters

1. The threshold voltage (V_{TH}) is defined as the gate voltage value when a drain current (I_{DS}) reaches 10^{-9} A with V_{DS}=0.1V.
2. The turn-off voltage (V_{OFF}) is defined as the gate voltage value when a drain current (I_{DS}) reaches 10^{-13} A with V_{DS}=10V.
3. The subthreshold slope (S.S.) is extracted from a 3-decades range in the sub-threshold region of the log_{10} (I_{DS}) *vs* V_G curve with V_{DS}=10V:

$$S.S. = \left[\frac{\partial log_{10}(I_{DS})}{\partial V_G}\right]^{-1} \quad \text{(S.1)}$$

4. The on-current (Ion) is the drain current while a TFT operates at V_{GS}=10V and V_{DS}=10V.
5. The on/off ratio is obtained by comparing between the on-current and the lowest current within the gate bias range of -25V to 10V.
6. The blocking voltage is defined as the highest drain bias that a TFT can sustain without a breakdown in the OFF state.
7. The shifts of threshold voltage in the thermal stability measurement

$$\Delta V_{TH}(ZnO, T)=V_{TH}(ZnO, T)-V_{TH}(ZnO, 300K) \quad \text{(S.2)}$$

$$\Delta V_{TH}(MZO, T)=V_{TH}(MZO, T)-V_{TH}(MZO, 300K) \quad \text{(S.3)}$$

$$\Delta V_{TH}(m\text{-}MZO, T)=V_{TH}(m\text{-}MZO, T)-V_{TH}(m\text{-}MZO, 300K) \quad \text{(S.4)}$$

Extracted electrical parameters of HVTFTs

1. Table S1. Electrical characteristics of ZnO, MZO, and m-MZO HVTFTs with the equivalent channel length L=10μm:

HVTFT Devices	on/off Ratio	V_{OFF} (V)	S.S. (V/Decade)
ZnO	6.1×10^{7}	-19.0	1.37
MZO	6.7×10^{7}	-19.0	1.24
m-MZO	3.5×10^{10}	-13.0	0.53

2. Table S2. Voltage-blocking capabilities and output characteristics of m-MZO HVTFTs with three different channel lengths (L=10μm, 15μm, 25μm):

L(μm)	Ion (A)	Blocking Voltage(V)	Maximum Drain Current (A)
25	3.3×10^{-6}	609	3.2×10^{-5} (at V_{DS}=200 V*)
15	5.8×10^{-6}	447	3.0×10^{-5} (at V_{DS}=110 V)
10	3.5×10^{-5}	305	1.3×10^{-4} (at V_{DS}=70 V)

*instrument limit
